# SHP-2 deletion in CD4Cre expressing chondrocyte precursors leads to tumor development with wrist tropism

**DOI:** 10.1038/s41598-021-99339-0

**Published:** 2021-10-08

**Authors:** Jeffrey T. McNamara, Kelsey E. Huntington, Samantha Borys, Chathuraka T. Jayasuriya, Laurent Brossay

**Affiliations:** 1 Division of Biology and Medicine, Department of Molecular Microbiology and Immunology, Brown University Alpert Medical School, Providence, RI 02912 USA; 2 Department of Orthopaedics, Rhode Island Hospital and Brown University Alpert Medical School, Providence, RI USA

**Keywords:** Bone cancer, Cartilage development, Differentiation, Osteoimmunology, Mechanisms of disease

## Abstract

Due to redundancy with other tyrosine phosphatases, the ubiquitously expressed tyrosine phosphatase SHP-2 (encoded by *Ptpn11*) is not required for T cell development. However, *Ptpn11* gene deletion driven by CD4 Cre recombinase leads to cartilage tumors in the wrist. Using a fate mapping system, we demonstrate that wrist tumor development correlates with increased frequency and numbers of non-hematopoietic lineage negative CD45 negative cells with a bone chondrocyte stromal cell precursor cell (BCSP) phenotype. Importantly, the BCSP subset has a history of CD4 expression and a marked wrist location tropism, explaining why the wrist is the main site of tumor development. Mechanistically, we found that in SHP-2 absence, SOX-9 is no longer regulated, leading to an uncontrolled proliferation of the BCSP subset. Altogether, these results identify a unique subset of chondrocyte precursors tightly regulated by SHP-2. These findings underscore the need for the development of methods to therapeutically target this subset of cells, which could potentially have an impact on treatment of SHP-2 dysfunction linked debilitating diseases.

## Introduction

Protein tyrosine phosphatase nonreceptor type 11 (*Ptpn11)* encodes for Src homology region 2 domain-containing phosphatase-2 (SHP-2) protein, a regulatory tyrosine phosphatase which, when globally knocked out, leads to embryonic lethality^1^. Interestingly, SHP-2 has been shown to have activating and inhibitory signaling roles. It activates extracellular-signal-regulated kinases (ERK) downstream of growth factor and cytokine receptor but inhibits T cell activation through programmed death receptor 1 (PD-1)^2,3^. SHP-2 has also been demonstrated to play an important role in embryonic hematopoietic stem cell (HSC) and mesenchymal stem cell (MSC) development and function^4^. Clinically, inherited mutations of *Ptpn11* can cause Noonan and Leopard syndrome, and mutations have been associated with numerous types of cancer including leukemia, breast, lung, liver, gastric, laryngeal, and oral cancers^5,6^. Loss of function mutations of SHP-2 cause metachondromatosis, a rare syndrome leading to cartilage tumors^7,8^. As SHP-2 acts in both stimulatory/inhibitory signaling within a variety of cell types and pathways, a clearer contextual understanding is needed of SHP-2 signaling roles based on cellular expression and how these relate to disease phenotype^9^.

In mice, conditional deletion of *Ptnp11* in chondrocytes has been shown to regulate terminal differentiation and growth plate architecture^10^. Additionally, conditional deletion of *Ptnp11* in chondrocytes demonstrated that SHP-2 plays a role in the osteogenic fate of hypertrophic chondrocytes^11^ and modifies SRY-related HMG box-containing (SOX) 9 expression^12^. Intriguingly, our lab demonstrated that SHP-2 deletion in CD4^+^ cells, a canonical T cell marker expressed in all αβ T cells during thymic development, leads to skeletal tumors in mice without impacting T cell development and function^13^. In support of these findings, mice deficient for SHP-2 downstream components, such as Ras guanine exchange factors (RasGEFs) SOS-1 and -2^14^ and extracellular signal related kinase (ERK)-1 and -2^15^ in CD4^+^ cells, also develop skeletal tumors. Together these studies suggest a possible link to chondrocyte development, which is yet to be definitively established.

Osteogenesis occurs through two major processes: intramembranous ossification, where mesenchymal tissue is directly transformed into bone, and endochondral ossification, where a cartilage intermediate is formed and replaced by bone cells^16,17^. Long bone development occurs via endochondral ossification in the growth plates developing through MSCs generated from the lateral plate mesoderm^17^. The growth plate contains chondrocytes, which are guided through tightly controlled stages of development from round cells in the resting zone, to columnar cells in the proliferative zone, and finally to larger cells in the pre-hypertrophic/hypertrophic zones where ossification occurs^18^. Chondrocytes develop from a progressively less multipotent cell lineage, including: MSCs, skeletal system stem cells (SSCs), bone chondrocyte stromal cell precursor cells (BCSPs), pre-chondrocyte cells (PCPs), resting chondrocytes, pre- and hypertrophic chondrocytes located within the end bones, and growth plates^18,19^. Chondrocyte development and regulation occurs through the transcription factor SOX-9, which is expressed in progenitor cells though hypertrophic chondrocytes^20^. Growth plate closure/fusion in humans occurs after sexual maturation and is triggered by the estrogen receptor. In mice, the estrogen receptor also triggers growth plate closure which is reduced in size in adulthood but never completely closed^21,22^.

To mark cells that have deleted the *Ptpn11* gene, we crossed SHP-2^fl/fl^-CD4-Cre mice to RosaYFP reporter mice. Using this mouse line, we demonstrated that wrist tumors are largely absent in SHP-2^fl/fl^-CD4-Cre mice at 1 month of age, develop at 3 months of age, and progress to larger tumors at 6 months of age. Tumor progression is correlated with increased frequency and numbers of non-hematopoietic Lin^−^CD45^−^YFP^+^ cells in the growth plates of the distal ulna and radius, which can still be identified within large tumor growth plate-like structures. Importantly, we also demonstrate that these cells fit into a BCSP phenotype, suggesting a defect within this developmental cell type. Notably, the CD45^−^YFP^+^ cells have a marked wrist location tropism potentially explaining why the wrist is the main site of tumor development. Finally, we showed that SHP-2 negatively regulates SOX-9 intrinsically in CD45^−^YFP^+^ BCSP-like cells.

## Methods

### Mice

SHP-2^fl/fl^-CD4-Cre-ROSA^EYFP^ mice were previously described^13^. The SHP-2^fl/fl^ mice were originally generated by Dr. Wentian Yang at the Rhode Island Hospital^23^. SHP-2^fl/fl^-CD4-Cre- ROSA^EYFP^ mice were crossed with B6.129S7-*Sox9*^*tm2Crm*^/J, purchased from Jackson Laboratories (Cat #:013106), to generate DKO SOX9^fl/fl^-SHP-2^fl/fl^-CD4-Cre mice. CD4-Cre was genotyped as described by Westendorf et al.^24^. To determine excision of the floxed allele the following primers were used, F 5′-TAGCTGCTTTAACCCTCTGTGT-3′, R1 5′-CATCAGAGCAGGCCATATTCC-3′ and R2 5′-TCACAATGAAGGTTCCTGTCC-3′.

### End bone cell isolation

For isolation of cells from the bone ends, a protocol was adapted from what has been previously described^25–27^. Briefly, the skin and soft tissue were removed from the ulna, radius, femur and tibia, and the bones were placed in cold PBS. Where applicable, gross images were taken of the bones with a ruler in frame. The end of each bone was then separated from the long bone at the epiphysis using scissors. The wrist end bone contained the distal ends of the ulna and radius. The pooled end bones contained the proximal ends of the ulna and radius (elbow) and the distal and proximal ends of the femur and tibia (hip, knee, ankle). The bone ends were incubated for 1 h at 37C in digestion buffer containing 1.5 mg/ml Collagenase D (Roche Diagnostics) and 0.125% Trypsin (HyClone) in RPMI (HyClone), then washed with 1% PBS-serum. After the first digestion, the bone ends were then cut into smaller pieces using a scalpel and incubated a second time for 1 h at 37C in fresh digestion buffer. The bones were washed in 1% PBS-serum, and then both digestions and washes were pooled. Cells were pelleted and characterized by flow cytometry.

### Spleen and bone marrow cell isolation

After removal of the end bones, the long bones were flushed with a syringe and needle with cold 1% PBS-serum. Bone marrow cells were then analyzed by flow cytometry. Spleens were dissociated in 1% PBS-serum, filtered, and underlayed with lympholyte-M (Cedarlane Laboratories).

### Antibodies and flow cytometry

Single cell suspensions were stained with fluorochrome-conjugated monoclonal antibodies and a 2.4G2 blocking antibody in the dark for 30 min on ice. Samples were run on a FACSAria III (BD Biosciences), and analyzed using FlowJo (Tree Star Inc). Maintenance of YFP expression during intranuclear staining was performed as previously described^28^. The antibodies listed below were used for flow cytometry: CD4-eF450 (Clone: GK1.5, eBioscience), CD29-APC-eF780 (Clone: BioHMb1-1, Invitrogen), CD45-BV570 (Clone: 30-f11, Biolegend), CD49f-PerCP-eF710 (Clone: BioGoH3, Invitrogen), CD51-PE (Clone: RMV-7, eBioscience), CD105-APC (Clone: MJ7/18, eBioscience), CD105-eF450 (Clone: MJ7/18, eBioscience), CD200-PerCP-eF710 (Clone: OX90, ebioscience), CD202b-AF647 (Clone: TEK, Biolegend), Lineage (CD3, B220, CD11b, GR-1, TER11-9)-PerCP-Cy5.5 (BD Biosciences), TER119-SuperBright645 (Clone: TER119, Invitrogen), Thy1.2 (CD90.2)-AF700 (Clone: 30-H12, Biolegend), and 6C3-Pe-Cy7 (Clone: 6C3, Biolegend). The following cellular subsets were sorted on the FACSAria III (BD Biosciences): splenic T cells (CD45^+^TCRβ^+^), splenic B cells (CD45^+^CD19^+^B220^+^), end bone chondrocytes (CD45^−^Lin^−^), end bone lymphocytes (CD45^+^Lin^+^), wrist chondrocytes (CD45^−^Lin^−^), wrist lymphocytes (CD45^+^Lin^+^).

### Immunofluorescent microscopy

Mice were euthanized at the indicated ages, and perfused with 4% PFA. The ulna, radius, femur, and tibia of each animal were collected, placed into 4% PFA for 2 h, and then washed with PBS. Bones were decalcified as previously described by placing the bones in 0.5 M EDTA for 24 h, then prepared for mounting by incubation in sucrose for 24 h^29^. Long bones were flash frozen in OCT (Fischer Healthcare), sectioned onto adhesive tape as previously described^30^, washed, blocked with 1% rat serum in PBS, and then stained. Staining antibodies used included: DAPI, TCRβ-PE (Clone: H57-597, eBioscience), and CD44-PE (Clone: IM7, eBioscience). After staining, the tissues were mounted with Fluoromount-G (Invitrogen) and imaged using a Zeiss Axiovert 200 M Fluorescence Microscope (Zeiss) in the Leduc Imaging Core (Brown University). Images were analyzed with FIJI^31^.

### Statistical analysis

Statistical analyses were performed with Prism 7.0 or 8.0 (Graph-Pad Software, Inc.). Unpaired two-tailed Student’s t tests were used to compare two individual groups. Error bars indicate SEM and *p < 0.05, **p < 0.01, ***p < 0.001 and ****p < 0.0001. For analysis of contingency table between tumor and non-tumor animals, Fisher’s-exact test was used.

### Ethics approval and consent to participate

All mouse experiments were carried out in accordance with the Guide for Care and Use of Laboratory Animals, as defined by the NIH (PHS Assurance #A3284-01). Animal protocols were reviewed and approved by the Institutional Animal Care and use Committee (IACUC) of Brown University. All animals were housed in a centralized and AAALAC-accredited research animal facility. The reporting in this manuscript follows the recommendations in the ARRIVE guidelines.

## Results

### SHP-2 KO mice develop tumors with a marked wrist location tropism

Using mice conditionally deficient for SHP-2 in the T cell lineage, we previously reported that the development and function of these lymphocytes is globally intact^13^. However, we also reported that in aging mice*, Ptpn11* gene deletion driven by CD4 Cre recombinase leads to wrist tumors in a T cell-independent manner^13^. To mark cells that have deleted the *Ptpn11* gene, we crossed the SHP-2^fl/fl^-CD4Cre mice to RosaYFP reporter mice^32^. For simplicity, we will refer to SHP-2^fl/fl^-EYFP^+^-CD4Cre animals as KO, and their littermate controls SHP-2^fl/–^EYFP^+^-CD4Cre as Het, throughout. We previously reported that tumors were observed in the wrists (> 3 months of age), and sporadically in the hips, knees, and vertebrae of older mice (> 5 months of age)^13^. Upon further examination, the tumors were mostly identified in bones formed by endochondral ossification, specifically long bones and vertebrae. We next monitored wrist tumor development at 1, 3, and 6 months of age. Tumors were absent in Het animals at each timepoint examined and mostly undetectable in 1-month-old KO animals (Supplemental Fig. 1A). At 3 months of age, wrist tumors were identified in all KO animals, and tumors continued to progress in size at 6 months of age, becoming visible in gross images (Supplemental Fig. 1A). When measured, wrists were significantly wider in 6-month-old KO animals as compared to Het animals (5.5 ± 0.5 mm versus 1.5 ± 0.4 mm; p < 0.05, Supplemental Fig. 1B). Tumor growth was multidimensional and observed in the distal head of the ulna and radius but not in the proximal ends (Supplemental Fig. 1A). Interestingly, tumors were not observed in the wrist carpels or meta carpels and did not develop along the shaft of the long bones. However, some of the animals developed non-wrist tumors in other joints (but not all), that were uni- and bilateral (Supplementary Fig. 1C). Additional tumors could be seen in the hip (proximal femur), and knee (distal femur and proximal tibia), but were never observed in the ankle (distal tibia and fibula) and feet (Supplementary Fig. 1C). In contrast to the wrist tumors, tumors in other joints were mostly small. Altogether, these data show that SHP-2 deletion in CD45^−^CD4^+^ cells (YFP^+^) lead to tumor development with a marked wrist location tropism.

**Figure 1:**
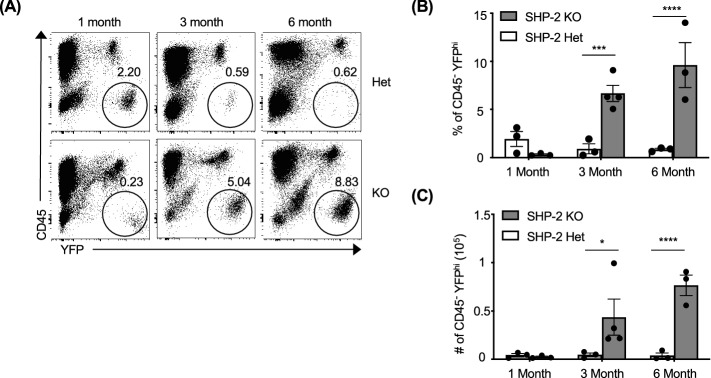
Increases in non-hematopoietic YFP^+^ cells correlate with progression of wrist tumors. (**A**) Representative staining of cells isolated from the wrists of Het and KO animals at 1, 3 and 6 months of age. (**B**) Frequency and (**C**) total number of CD45^−^YFP^+^ cells (black circled population in panel (**A**) isolated from the wrists at each time point (n = 3–4). Data are from a single experiment and represent mean ± SEM. *p < 0.05, **p < 0.01, ***p < 0.001, and ****p < 0.0001. See also Figure S1.

### Non-hematopoietic CD45^−^YFP^+^ cell number increased during wrist tumor development

We previously demonstrated that *Ptpn11* gene deletion driven by CD4 Cre recombinase leads to cartilage tumors in a T cell independent manner since the phenotype is observed even in RAG-1 absence^13^. However, the cell subset responsible for this phenotype was not characterized. To investigate this, we first examined cells from the contralateral wrists of 1-, 3- and 6-month-old Het and KO animals. Surprisingly, we identified a non-hematopoietic CD45^−^YFP^+^ cell subset which progressively increased in frequency and number in the wrists of KO animals as they aged (Fig. 1). These increases were not present in Het animals and became significant between the two groups at 6 months of age (p < 0.05). Significant increases, of a lesser magnitude, in percentage and number of CD45^−^YFP^+^ cells were also identified in the pooled bone ends of 6-month-old KO animals as compared to Het animals (Supplemental Fig. 2A,B). These changes were not identified in the central bone marrow or spleen, where only very low frequencies (< 0.05%) were identified at any timepoint in Het or KO animals (Supplemental Fig. 2C,D).

**Figure 2:**
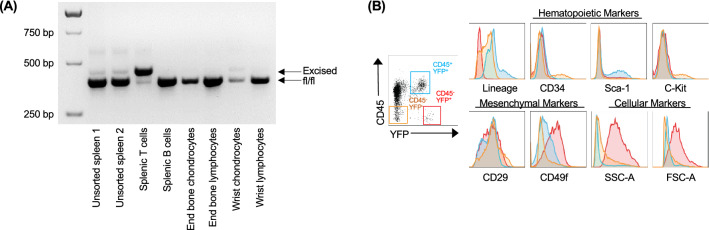
Genetic excision of the floxed region of the *Ptpn11* gene in tumor-associated CD45^−^YFP^+^ cells, which have a non-hematopoietic, mesenchymal phenotype. (**A**) Cells from SHP-2 KO spleens, end bones, and wrist of two 3–4 week old mice were sorted and pooled. A PCR was performed to examine the excision of the floxed site, where the smaller band represents the non-excised floxed band, and the upper band is the excised floxed band. Lanes from left to right: unsorted splenocytes from mouse 1 and 2, sorted splenic T cells (CD45^+^TCRβ^+^), sorted splenic B cells (CD45^+^CD19^+^B220^+^), end bone chondrocytes (CD45^−^Lin^−^), end bone lymphocytes (CD45^+^Lin^+^), wrist chondrocytes (CD45^−^Lin^−^), wrist lymphocytes (CD45^+^Lin^+^). Data is from cells isolated from SHP-2 KO animals from two independent experiments. (**B**) Representative histograms of hematopoietic markers (lineage cocktail (CD11b, TER119, LY-6C, B220), CD34, Sca-1, C-Kit) and mesenchymal markers (CD29, CD49f), and cellular phenotype by side scatter (SSC-A) and forward scatter (FSC-A). For histograms, CD45^−^YFP^+^ cells (red lines), CD45^−^YFP^−^ (orange lines), and CD45^+^YFP^+^ (blue lines) cells were plotted (n = 6). Data is from cells isolated from 5 to 6-month-old SHP-2 KO animals from two independent experiments.

### CD45^−^YFP^+^ cells isolated from tumors have a mesenchymal phenotype

We performed an extensive characterization of the CD45^−^YFP^+^ cells associated with the development of wrist tumors. Following cell sorting of different cell subsets of SHP-2^fl/fl^-EYFP^+^-CD4Cre mice, we performed a PCR to determine whether genetic excision of the floxed region of the *Ptpn11* gene can be observed. In B cells only the floxed allele was detected (Fig. 2A). As expected, in sorted T cells, the excised allele was predominant. Importantly, while end bone chondrocytes displayed no excision, both the *Ptpn11* floxed and excised alleles were detected in wrist chondrocytes, indicating in some of these chondrocytes *Ptpn11* has been deleted (Fig. 2A). Interestingly, the CD45^−^YFP^+^ tumor-associated cells were found to be negative for all hematopoietic markers examined (Fig. 2B). This included a lineage cocktail (Ter119 (erythrocytes), LY6C (granulocytes), CD11b (macrophages), and B220 (B-cells)), CD34, and C-Kit, while few expressed Sca-1. Notably, these cells expressed the mesenchymal markers integrin β1 (CD29) as well as integrin β6 (CD49f), and were found to have significantly greater forward scatter and side scatter compared to CD45^−^YFP^−^ and CD45^+^YFP^+^ cells (Fig. 2B). Taken together, these data indicate that the CD45^−^YFP^+^ cells are derived from mesenchymal origin.

### CD45^−^YFP^+^ cells have an arrested stem cell phenotype

**Figure 3:**
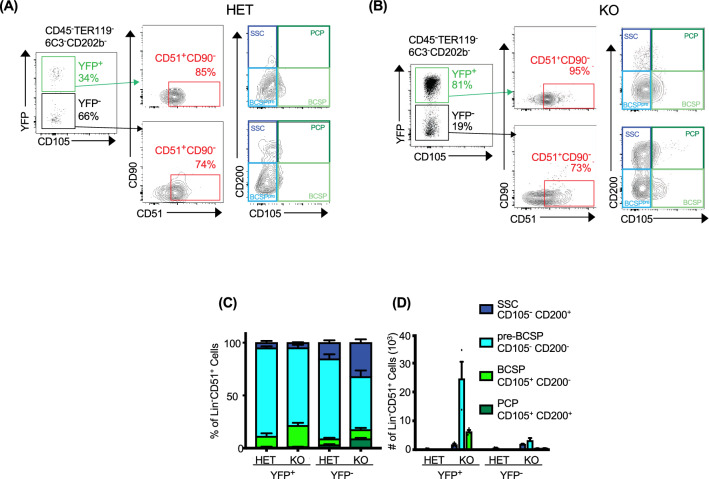
Increased YFP^+^ tumor-associated cells have a bone chondrocyte stromal precursor (BCSP) cell phenotype. (**A**,**B**) Representative flow cytometry gating strategy for identifying cells isolated from adult wrists in Het and KO animals. Lineage negative cells (CD45^−^, TER119^−^, 6C3^−^, CD202b^−^) were first separated into YFP^+^ and YFP^−^ cell populations (green and black boxes, respectively). Each subset was then gated by CD51 (Alpha V integrin) and CD90 (Thy1.1) with CD51^+^CD90^−^ cells (red box) being further subdivided into skeletal system stem cell phenotypes by CD105 and CD200 with the following assignments: SSC (CD105^−^CD200^+^, dark blue), pre-BCSP (CD105^−^CD200^−^, light blue), BCSP (CD105^+^CD200^−^, green), PCP (CD105^+^CD200^+^, dark green). Percentage (**C**) and total number (**D**) of each skeletal system cell phenotypes for YFP^+^ and YFP^−^ cells within both SHP-2 Het and KO animals (n = 3). Data are taken from one of two independent experiments and represent mean ± SEM. *p < 0.05, **p < 0.01, ***p < 0.001, and ****p < 0.0001.

Having determined that the tumor-associated CD45^−^YFP^−^ cells were from mesenchymal origin, we then continued the characterization of these cells. Phenotypic expression of SSC markers has been used to identify chondrocyte precursor cells^19^. Wrist cells from adult (3–5 months of age) Het and KO animals were extensively phenotyped to more accurately define the cells associated with tumors (Fig. 3). First, lineage negative cells (CD45^−^, TER119^−^, 6C3^−^, CD202b^−^) were divided into YFP^+^ and YFP^−^ subsets. While approximately 2/3 of the lineage negative cells isolated were YFP^−^ in Het mice (Fig. 3A), the inverse was found in KO animals with approximately 4/5 of the cells being YFP^+^ (Fig. 3B). Lin^−^YFP^+^ and Lin^−^YFP^−^ subsets isolated from Het and KO animals were mostly CD51^+^CD90^−^ (73–95%), suggesting an SSC origin and disruption of developmental progression. Both YFP^+^ and YFP^−^ cells were examined for the expression of markers according to the following progressive development schema; SSC (CD200^+^CD105^−^), pre-BCSP Cell C (CD105^−^CD200^−^), BCSP (CD105^+^CD200^−^), and PCP (CD105^+^CD200^+^). When Lin^−^YFP^+^ cells from tumors were further characterized using anti-CD105 and anti-CD200 mAbs, we found that this subset was largely composed of a pre-BCSP (CD105^−^CD200^−^) or BCSP (CD105^+^CD200^−^) phenotype with few cells expressing a SSC (CD200^+^CD105^−^) and almost none expressing a PCP (CD105^+^CD200^+^) phenotype (Fig. 3C,D, left two columns). Within the Lin^−^YFP^−^ cell population, larger percentages of SSC and PCP cells were identified (Fig. 3C,D, right two columns). Importantly, YFP^+^ cells never progressed to the PCP phenotype and few CD90^+^YFP^+^ cells were identified. Altogether, these data demonstrate that CD45^−^YFP^+^ cells are mostly negative for lineage markers and express markers associated with mesenchymal stem cells and chondrocyte subsets. We concluded that YFP^+^ tumor associated cells have a BCSP cell phenotype.

### YFP^+^ cells are observed in the growth plate of both Het and KO young animals

**Figure 4:**
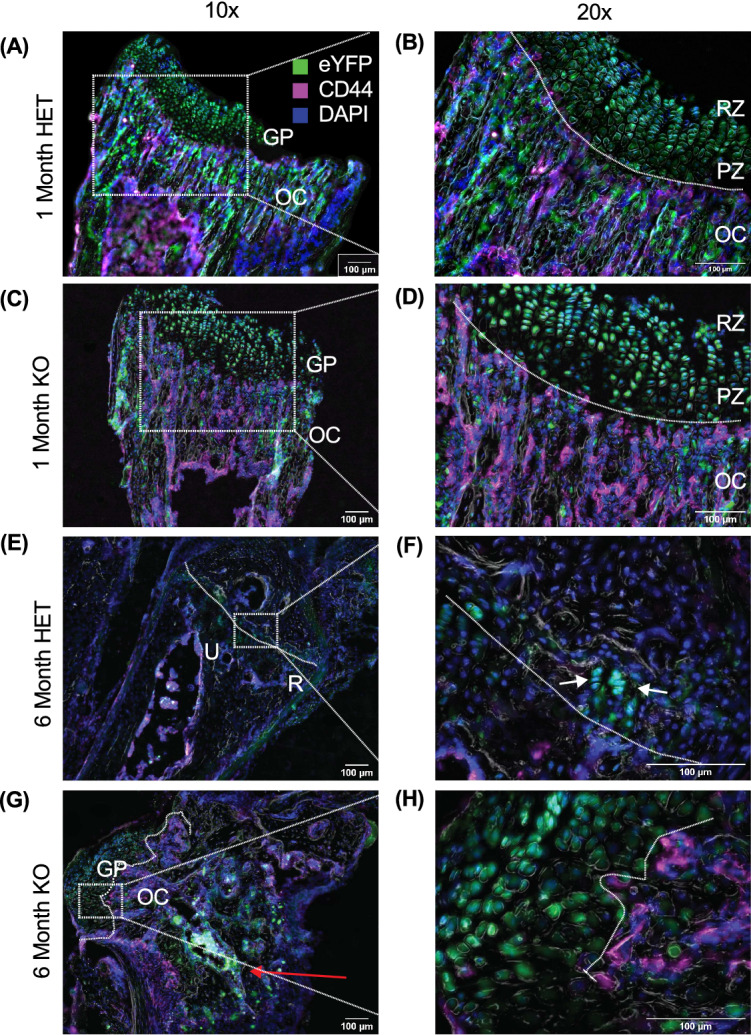
The growth plates of adolescent and adult KO mice contain YFP^+^ cells in wrist tumors. (**A**–**D**) Representative images of the distal forelimb from adolescent 1-month-old Het (**A**,**B**) and KO (**C**,**D**) animals. (**E**–**H**) Representative images from the same region taken from adult 6-month-old Het (**E**,**F**) and KO (**G**,**H**) animals. Low power shown in the left column (×10; scale bar 100 μm) with a high power inset (×20; scale bar 50 μm) marked by white dotted square and white dotted lines is shown in the right column. Additional white dotted lines indicate chondro-osseous junction where the ossification center (OC) is identified by CD44^+^ cells. The proliferative zone (PZ) of the growth plate (GP) contains the columnar chondrocytes whereas the resting zone (RZ) contains the round resting chondrocytes. *GP* Grow Plate, *Green* eYFP, *Blue* DAPI, *magenta* CD44, *gray* Phase Contrast. Forelimbs from n = 3 (6 total bones) animals were examined for each genotype at each timepoint.

Immunofluorescent images of 1-month-old animals revealed the presence of YFP^+^ in the growth plates of the distal ulna and radius in the KO animals as expected, as well as the heterozygote animals (Fig. 4). YFP^+^ cells were identified in both the resting and proliferative zones (PZ) of the growth plate (GP) which were marked by the chondrocyte columns. The chondro-osseous junction was delineated with an anti-CD44 mAb, which stains osteocytes and hypertrophic chondrocytes^33^ located beneath the chondrocyte columns. The YFP^+^ cells were only sporadically observed in the hypertrophic zone/ossification center (OC). In agreement with the flow cytometry, the YFP^+^ cells found in the growth plate did not co-stain with CD45 or TCRβ, whereas the YFP^+^ cells found in the ossification center were largely CD45^+^ and TCRβ^+^ (data not shown). Overall, the presence and location of these cells in the growth plates of KO and Het animals were found to be comparable at 1 month of age (Fig. 4A–D). YFP^+^ cells were also identified in the growth plates of other bones examined, with less frequency but comparable location. In 1-month-old KO animals, YFP^+^ cells were found in the chondrocyte columns of the growth plate within the distal femur but were more diffuse than the distal ulna or radius (Supplemental Fig. 3). Few to no YFP^+^ cells were identified in the proximal ulna or radius (elbow) of 1-month-old animals examined (data not shown). These data demonstrate that the YFP^+^ cells in adolescent Het and KO animals are found in comparable locations in normally structured growth plates.

### YFP^+^ cells are still present in growth plate-like structures of 6-month-old SHP-2 KO animals

YFP^+^ cells were identified in the remnants of the growth plate and still aligned in a columnar fashion in 6-month-old Het animals (Fig. 4E,F). It was noted that the columns contained far fewer cells as compared to 1-month-old animals. In addition, ossification centers marked by CD44 were not observed in 6-month-old Het animals. These animals had disorganized end bone architecture within the tumor. There were still active growth plate-like structures identified throughout the tumors of KO animals, which were marked by YFP^+^ cells in chondrocyte columns that appeared to be multidirectional (Fig. 4E,F). Importantly, an erratic chondro-osseous junction was noted at the base of the columns where CD44^+^ cells were still present in all 6-month-old KO tumors examined. Notably, large numbers of YFP^+^ cells were associated with the multi-directional growth plates but largely absent from ossification centers (Fig. 4G,H). To examine the presence of T cells in the tumors, sections were stained with an anti-TCRβ mAb. TCRβ^+^YFP^+^ cells were not observed in the growth plate-like areas, but clusters of T cells were seen beneath the ossification centers (Supplemental Fig. 4).

### Wrist tumor development mediated by SHP-2 deletion in CD4Cre expressing chondrocytes is SOX-9 dependent

**Figure 5:**
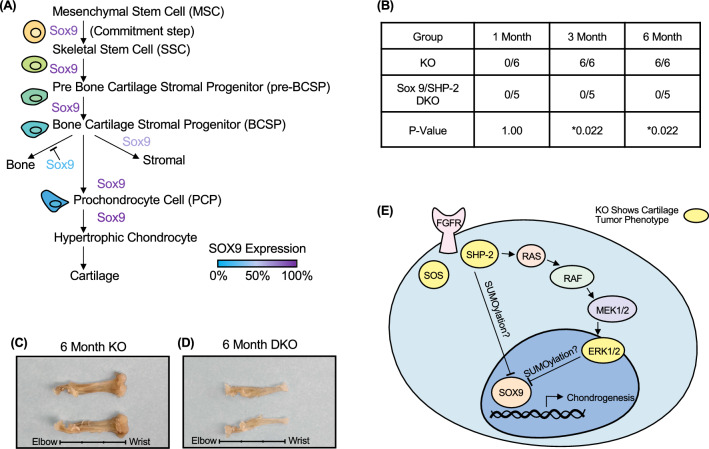
Chondrocyte tumor development is dependent on SOX-9. (**A**) Sox-9 expression in skeletal stem cell and chondrocyte development. (**B**) Gross observable wrist tumors from SHP-2 KO and SOX-9-SHP-2 KO animals at 1, 3, and 6 months of age. (**C**) SHP-2 KO and (**B**) SOX-9-SHP-2 KO representative images from adult 6-month-old forelimbs. (**D**) Graphical representation of chondrocyte SHP-2 SOX-9 relationship with a proposed role of fibroblast growth factor receptor (FGFR).

SOX-9 is required in several successive steps of the chondrocyte differentiation pathway during endochondral bone formation in vivo, to ensure chondrocyte differentiation and prevent development towards osteoblastic differentiation (Fig. 5A). Therefore, SOX-9 is absolutely necessary for chondrocyte specification and early differentiation^34^. We hypothesized that SHP-2-deficient cells may either differentiate into chondrocyte-like cells or cause neighboring SHP-2-sufficient chondrocytes to contribute to exostoses and enchondromas via paracrine signals. To test this hypothesis, we generated SOX-9^fl/fl^-SHP-2^fl/fl^CD4Cre (DKO) mice and SOX-9^fl/+^-SHP-2^fl/fl^CD4Cre control mice. In the SOX-9 heterozygote controls (SOX-9^fl/+^-SHP-2^fl/fl^-CD4Cre), one copy of *SOX9* is expressed, but *Ptpn11* is deleted from CD4^+^ cells. As shown in Fig. 5B,C, 100% of these mice developed tumors, indicating that expression of one copy of the *Sox9* gene is sufficient for the phenotype. In contrast, none of the double deficient animals (Sox9^fl/fl^-SHP-2^fl/fl^-CD4Cre) developed tumors (Fig. 5B,C). This suggests that SHP-2 intrinsically regulates SOX-9 expression in cells with a history of CD4 expression, and that SOX-9 expression is “unleashed” in the absence of SHP-2 (see model, Fig. 5E).

## Discussion

Cartilaginous tumors are ​common primary bone tumors​ that are a consequence of mutation in a large number of genes, including *Ptpn11*^8^. Several years ago, we reported that in aging mice, *Ptpn11* gene deletion driven by Cre recombinase under the control of CD4 promoter leads to wrist joint abnormalities in a T cell-independent manner. In this study, we aimed to identify the cell subset(s) responsible for the phenotype observed. Using a fate mapping system, we observed that the YFP^+^ cells were largely found in the chondrocyte columns where resting and proliferating chondrocytes reside. These data demonstrate that skeletal development occurs normally, and that the absence of SHP-2 in these cells causes tumors after formal bone development. When the tumors were examined by immunofluorescence, large erratic growth plates containing numerous YFP^+^ cells were observed in what appeared to be chondrocyte columns associated with an erratic ossification center. These data suggest that SHP-2 deletion in these cells causes the growth plates to remain active with the effected cells either over proliferating, transitioning into osteoblasts, or undergoing apoptosis. These changes in growth plate architecture are reminiscent of a specific SHP-2 deletion in chondrocytes^10^. Interestingly, the CD45^−^ lineage cells associated with the tumors were found to express high expression of YFP compared to CD4^+^ T cells. These cells were also shown to be larger (increased FSC), which may account for the slightly higher YFP MFI. Importantly, using the same CD4-Cre mice, others have shown that low levels of CD4 can be detected in cells isolated from cartilage^14^. Using a reporter system, we demonstrated that the earliest precursor cell subset to express YFP was a Skeletal Stem Cell (SSC), but it is possible that CD4 expression occurs earlier in the development process. Utilizing our fate mapping system may allow for a more in-depth investigation of wrist development processes.

Other conditional CD4 lineage specific knock-out models have demonstrated similar metachodromatosis (Fig. 5E, yellow) like phenotypes^14,15^. CD4 lineage cells were associated with tumor development at 24 weeks of age when ERK-1/2 were conditionally deleted in CD4 lineage cells, with tumor development being accelerated in the absence of T cells^15^. SOS1/2 deletion in CD4 lineage cells displayed tumor development when the mice aged^14^. Notably, the phenotype of our model is universal and more severe with all animals having grossly identifiable wrist tumors by 12 weeks of age and tumors necessitating euthanasia by 32 weeks of age. As SHP-2 is upstream of both ERK1/2 and SOS1/2, this suggests the presence of downstream regulatory mechanisms mitigating the potency of SOS and ERK phenotypes. Interestingly, when SHP-2 was specifically deleted from chondrocytes, cartilage defects were observed but were not reported to be specific to wrists^10^, suggesting that this subset of non-hematopoietic cells has a long bone articular growth plate tropism and possibly a forelimb specificity^10,35^. This is supported by the fact that even the heterozygote animals (1-month-old) have a larger percentage of YFP^+^ cells associated with the wrists as compared to other end bones examined. Altogether, these data demonstrate that a subset of chondrocyte precursors with a history of CD4 expression reside preferentially in the wrist.

Importantly, we have been able to isolate and characterize the YFP^+^ cells associated with tumor development. These cells are neither hematopoietic (CD45^−^CD11b^−^LY6C^−^B220^−^TER119^−^CD3e^−^, and TCRβ^−^) nor non-hematopoietic (CD34^−^, C-KIT^−^, and Sca-1^−^) stem cells. Integrins have been shown to be important in chondrocytes and chondrocyte development, where CD49f has been shown to be a marker of stemness in chondrocytes and mesenchymal stem cells. Normal chondrocytes have been shown to express CD51 and CD29 while arthritic chondrocytes express alpha6beta1 (CD49f/CD29)^36^. Non-immune lineage negative YFP^+^ cells extracted from tumors expressed mesenchymal stem cell markers (CD29^+^ and CD49f^+^), and together with the expression of additional markers (CD51^+^, CD90^−^, CD105^+/−^, and CD200^+/−^), can be categorized as bone chondrocyte stromal cell precursor cells (BCSP), which arise from MSCs (Figs. 3, 5). Interestingly, CD49f has also been shown to mark an injury induced activated BCSP subset that has a higher proliferative capacity and enhanced osteogenic potential^37^. It is therefore tempting to speculate that tumor development may be linked to joint healing dysregulation mediated by CD49f^+^ BCSP.

We previously hypothesized that the SHP-2-deficient cell subset may either inappropriately differentiate into chondrocyte-like cells or cause neighboring SHP-2-sufficient cells to contribute to exostoses and enchondromas via paracrine signals^13^. Bowen and colleagues showed that mosaic inactivation of *Ptpn11* in growth plates affect *Ptpn11* deficient and WT chondrocytes. We cannot exclude that SHP-2-deficient YFP^+^ cells may indirectly activate mesenchymal stem cells to undergo chondrogenesis. However, our data minimally demonstrate that SHP-2 intrinsically regulates SOX-9 expression in YFP marked cells, reinforcing the critical tumor suppressor role of SHP-2 in a subset of chondrocyte-like cells. This in agreement with a recent study showing that SHP-2 regulates osteochondroprogenitor fate determination via the phosphorylation and SUMOylation of SOX9^12^.

Fibroblast growth factor receptor 3 (FGFR-3) is a critical regulator of chondrocyte development and is upstream of SHP-2, ERK1/2 and SOS1/2. Inducible deletion of FGFR3 in adult mice resulted in endochondromas and osteochondromas, whereas global deletion of FGFR3 and one of its ligands, FGF18, led to aberrant chondrocyte proliferation in growth plates^38–40^. The conditional knockout also demonstrated that FGFR3 signaling was important in determining the polarity of chondrocytes, which is disrupted in the remaining chondrocyte columns identified in the tumors^38^. Interestingly, FGF18 and FGFR3 have been shown to have increased mRNA expression in SSC and BCSP cells^19^. It is therefore tempting to speculate that SHP-2 deletion in CD4^+^ cells leads to aberrant FGFR3 signaling and increased production of FGF18, which could account for the increased number of YFP^+^ and YFP^−^ SSC subsets associated with the tumors. This warrants further investigation.

## Conclusions

Taken together, using a fate mapping system, we demonstrate that wrist tumor development correlates with increased frequency and numbers of non-hematopoietic CD45^−^YFP^+^ cells with a BCSP phenotype. The YFP^+^ BCSP subset is present in the growth plates of the distal ulna and radius of both SHP-2 heterozygote and knockout mice. In the absence of SHP-2, SOX-9 is no longer regulated, leading to an uncontrolled proliferation of the YFP^+^ BCSP subset. This subset of chondrocyte precursors appears to be mostly localized in the wrist, and its dysregulation has serious consequences in this joint, possibly due to a mosaic effect as previously suggested^10^. Nonetheless, development of methods such as gene editing to therapeutically target this subset of cells could potentially have an impact on treatment of SHP-2 dysfunction linked debilitating diseases.

## Supplementary Information

Supplementary Information.
