# Supplementary material for: SHP-2 deletion in CD4Cre expressing chondrocyte precursors leads to tumor development with wrist tropism
Source: Sci Rep. 2021 Oct 8;11:20006. doi: 10.1038/s41598-021-99339-0 (PMC8501018; doi:10.1038/s41598-021-99339-0)
Supplement: Supplementary file 1 — Supplementary Information. [file 41598_2021_99339_MOESM1_ESM.pdf]

Supplemental Figure 1

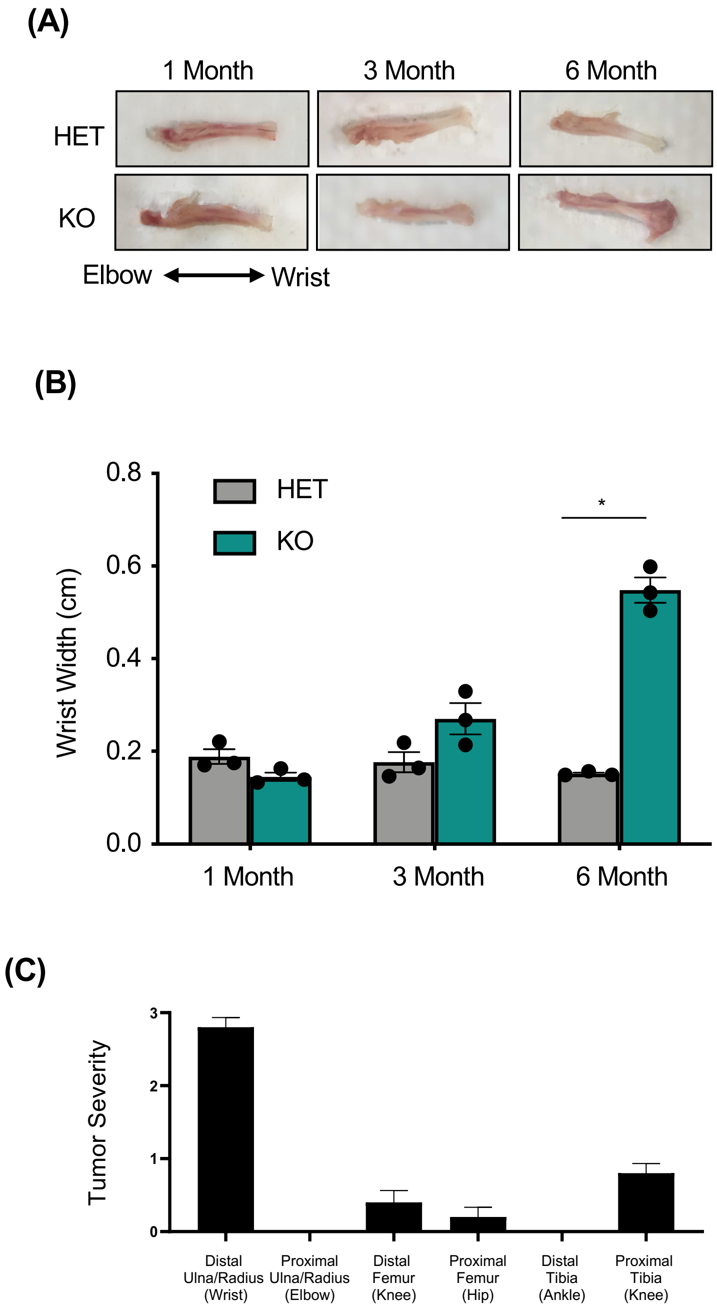

Prevalence	10/10	0/10	4/10	2/10	0/10	8/10
Percentage	100%	0%	40%	20%	0%	80%

Supplemental Figure 1. Progression of wrist tumors in SHP-2 KO animals. (A) Representative macroscopic images of contralateral forelimb taken from 1, 3 and 6 month old SHP-2 Het and SHP-2 KO animals. (B) Wrist widths of SHP-2 Het (grey bars) and SHP-2 KO (green bars) measured in imageJ from macroscopic images for each timepoint (n=3-4 error bars; error bars = SEM). (C) Prevalence and severity of tumors on each long bone end. Each end of the bone (right and left) was examined for tumor development and graded as 0= no tumor, 1 = slightly detectable, 2 = moderate easily detectable, 3 = severe grossly observable. Graph represents mean score of the bone ends and table represents prevalence and percentage detected (n=5).

Supplemental Figure 2

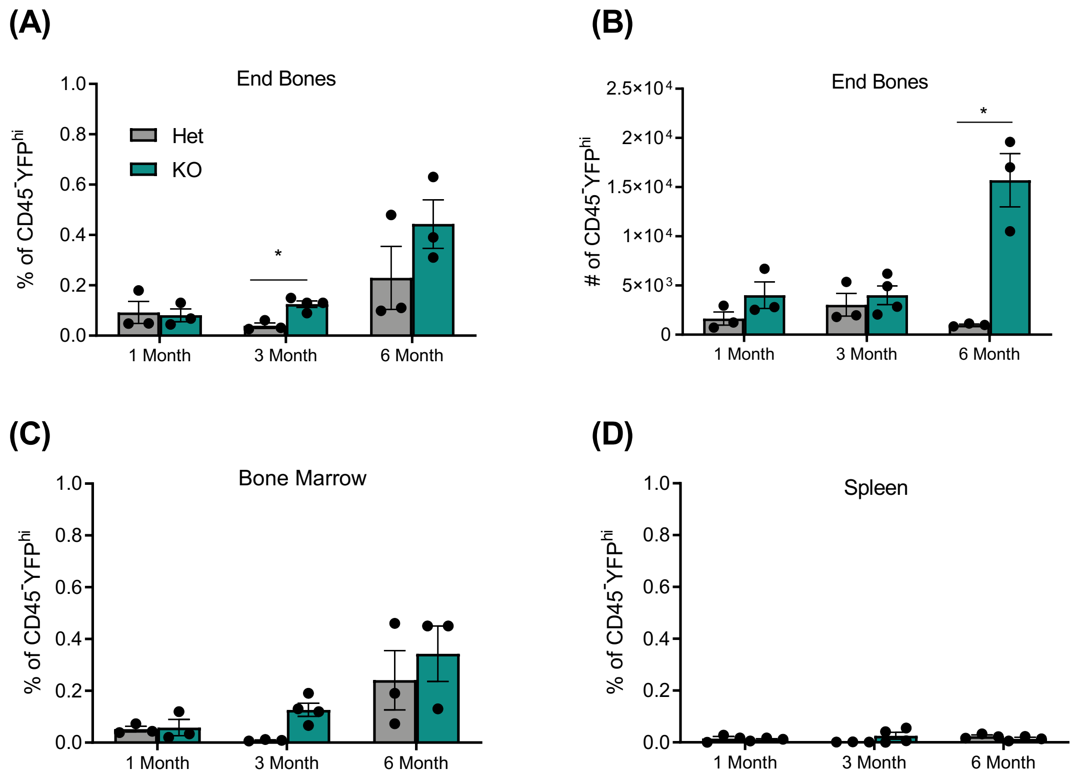

Supplemental Figure 2. Increased frequency of non-hematopoietic YFP+ cells Identified in in bone ends but not in peripheral compartments. (A) Frequency and (B) number of CD45-YFP+ cells identified in pooled bone ends (pooled proximal and distal femur, proximal and distal tibia and proximal forelimb). (C) Frequency bone marrow and (D) spleen of 1, 3, and 6 month old SHP-2 Het and SHP-2 KO animals (n=3-4, error bars = SEM).

Supplemental Figure 3

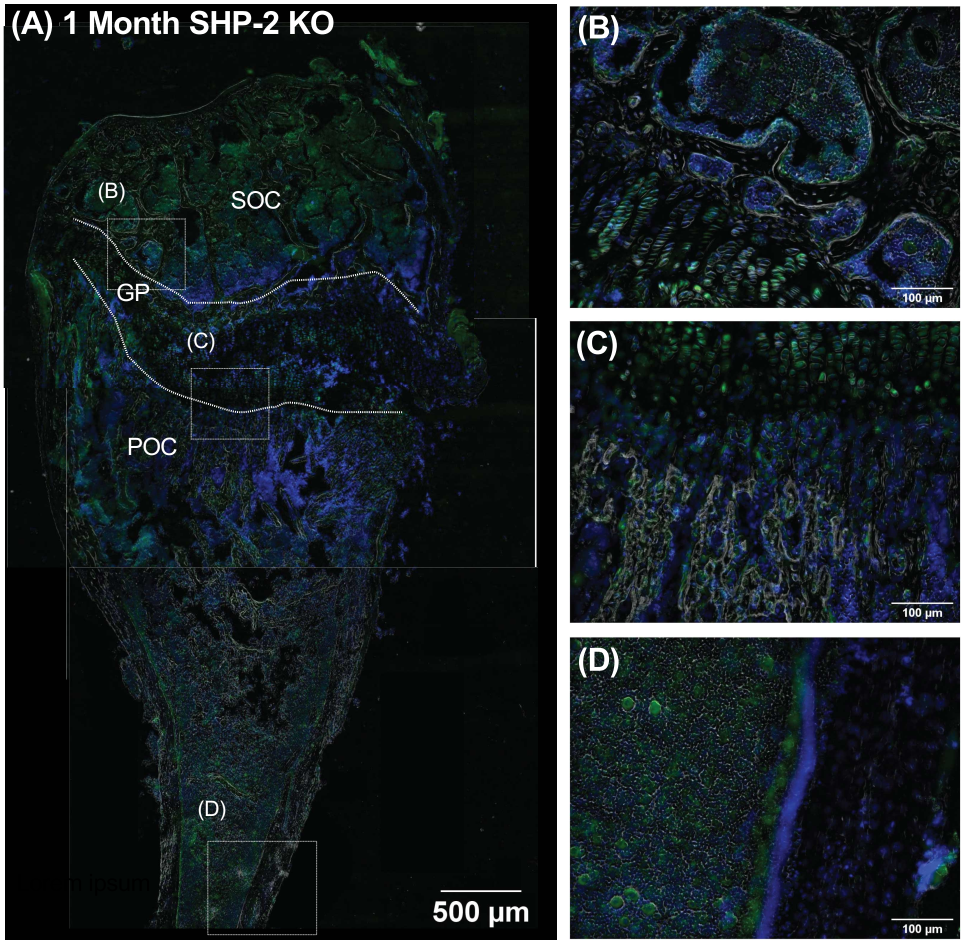

Supplemental Figure 3. Diffuse YFP^{+} cells identified in the femur of 1 month SHP-2 KO animals. (A) Low power (5x) stitched image of 6 month SHP-2 KO distal head of the femur. (B) 20x zoom of distal growth plate, (C) proximal growth plate and (D) central bone cavity. gray= phase contrast, green = eYFP, blue= DAPI

Supplemental Figure 4

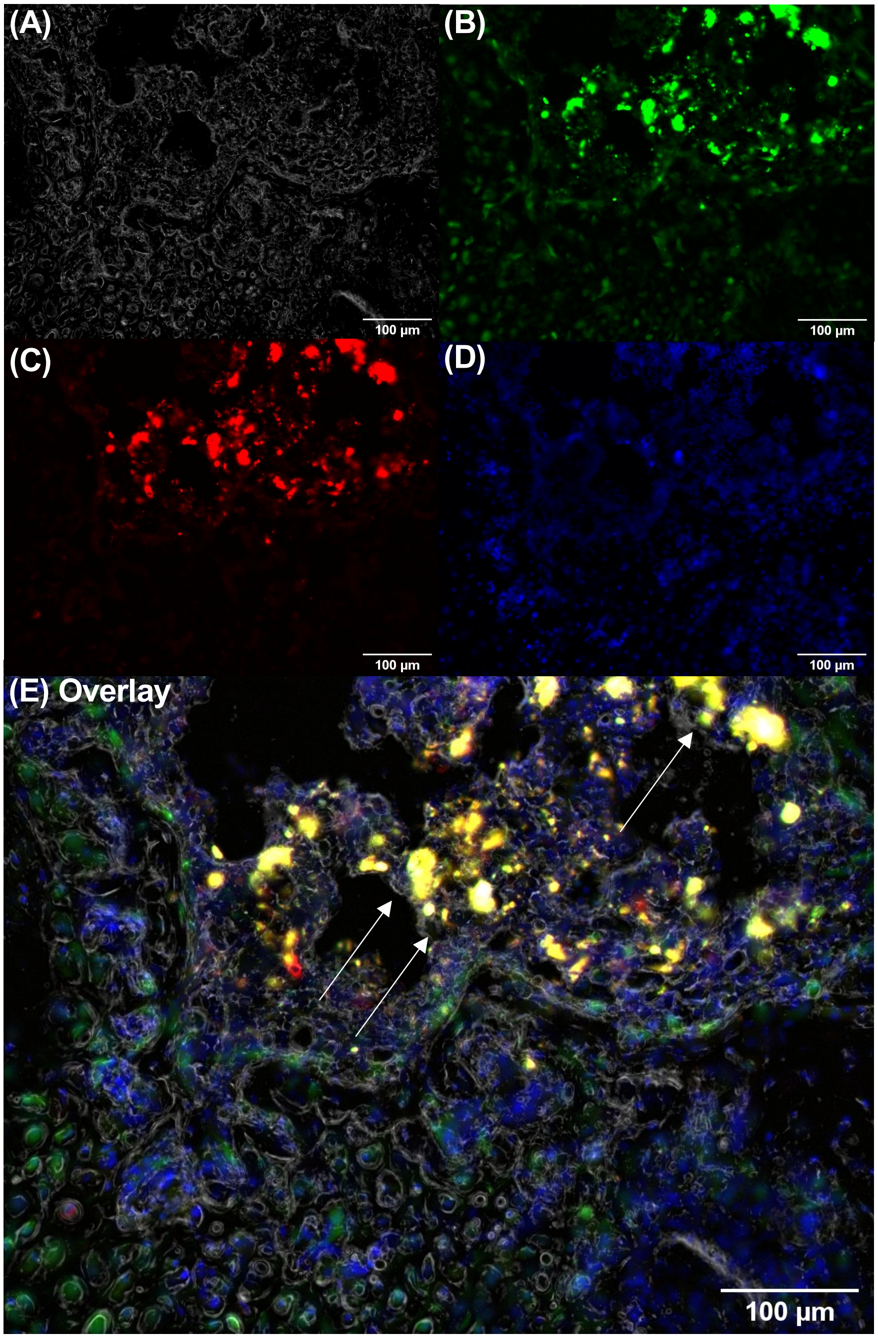

Supplemental Figure 4. T-cell clusters identified proximal to aberrant growth plates in SHP-KO wrists. (A) 20x phase (gray), (B) eYFP (green), (C) TCRβ (red), (D) DAPI (blue) and (E) overlay of wrist tumor of a 6 month SHP-KO animal. Yellow cells (indicated by white arrows) represent eYFP (CD4-Cre^{+}) TCRβ dual stained cells.
